# Oxidative muscles have better mitochondrial homeostasis than glycolytic muscles throughout life and maintain mitochondrial function during aging

**DOI:** 10.18632/aging.101643

**Published:** 2018-11-18

**Authors:** Annunziata N. Crupi, Jordan S. Nunnelee, David J. Taylor, Amandine Thomas, Jean-Philippe Vit, Celine E. Riera, Roberta A. Gottlieb, Helen S. Goodridge

**Affiliations:** 1 Board of Governors Regenerative Medicine Institute, Cedars-Sinai Medical Center, Los Angeles, CA 90048, USA; 2 Department of Biomedical Sciences, Cedars-Sinai Medical Center, Los Angeles, CA 90048, USA; 3 Smidt Heart Institute and Barbra Streisand Women's Heart Center, Cedars-Sinai Medical Center, Los Angeles, CA 90048, USA; 4 Biobehavioral Research Core, Cedars-Sinai Medical Center, Los Angeles, CA 90048, USA; 5 Center for Neural Science and Medicine, Cedars-Sinai Medical Center, Los Angeles, CA 90048, USA; * Equal contribution

**Keywords:** sarcopenia, mitochondria, glycolytic, oxidative, skeletal muscle, aging

## Abstract

Preservation of mitochondrial function, which is dependent on mitochondrial homeostasis (biogenesis, dynamics, disposal/recycling), is critical for maintenance of skeletal muscle function. Skeletal muscle performance declines upon aging (sarcopenia) and is accompanied by decreased mitochondrial function in fast-glycolytic muscles. Oxidative metabolism promotes mitochondrial homeostasis, so we investigated whether mitochondrial function is preserved in oxidative muscles. We compared tibialis anterior (predominantly glycolytic) and soleus (oxidative) muscles from young (3 mo) and old (28-29 mo) C57BL/6J mice. Throughout life, the soleus remained more oxidative than the tibialis anterior and expressed higher levels of markers of mitochondrial biogenesis, fission/fusion and autophagy. The respiratory capacity of mitochondria isolated from the tibialis anterior, but not the soleus, declined upon aging. The soleus and tibialis anterior exhibited similar aging-associated changes in mitochondrial biogenesis, fission/fusion, disposal and autophagy marker expression, but opposite changes in fiber composition: the most oxidative fibers declined in the tibialis anterior, while the more glycolytic fibers declined in the soleus. In conclusion, oxidative muscles are protected from mitochondrial aging, probably due to better mitochondrial homeostasis *ab initio* and aging-associated changes in fiber composition. Exercise training aimed at enriching oxidative fibers may be valuable in preventing mitochondria-related aging and its contribution to sarcopenia.

## Introduction

Skeletal muscles comprise fibers with a range of contractile properties and metabolic needs. Fast contractions rely on glycolysis, which occurs in the cytosol and permits rapid ATP generation, but is inefficient. Slow contractions allow ATP production by mitochondrial oxidative phosphorylation, which is slower, but more efficient. Each muscle comprises a distinct combination of fast-glycolytic, fast-oxidative/glycolytic, and slow-oxidative fibers [1,2]. The oxidative capacity of muscle fibers is determined by their mitochondrial content.

Mitochondrial function declines in the aged skeletal muscle of several species, including humans [3–11]. Mitochondrial biogenesis, dynamics (fission/fusion) and recycling are essential for maintenance of mitochondrial function, and aging is associated with impaired mitochondrial maintenance (reviewed in [6,12]). Indeed, mitochondrial morphology, an indicator of fission/fusion balance, has been shown to change in aged mouse muscles [13], and skeletal muscle aging is associated with altered mitochondrial dynamics and quality control in mice [14]. Moreover, improving mitochondrial homeostasis/function has been demonstrated to rejuvenate several tissues in various species, resulting in increased longevity [12].

Factors that regulate mitochondrial homeostasis are differentially expressed according to fiber type [2,15–17]. A higher rate of mitochondrial turnover might preserve mitochondrial function, and thus cell function, during aging [18]. In fact, strategies that improve mitochondrial biogenesis, such as overexpression of PGC-1α [19] or PGC-1β [2], improve exercise performance [2] and protect against muscle atrophy [19]. Moreover, mitochondrial autophagy (mitophagy), which selectively recycles damaged mitochondria, is fundamental for mitochondrial disposal, turnover and biogenesis [12,20], and enhancing dependence on oxidative phosphorylation was shown to increase mitophagy and clearance of mtDNA mutations [21]. However, the literature regarding the aging-associated changes in mitochondrial function and homeostasis in glycolytic and oxidative muscles is somewhat controversial [3–10].

Previous reports have demonstrated aging-associated changes in mitochondrial function and homeostasis in skeletal muscles, including muscle type-specific differences [13,22], but to the best of our knowledge, the intrinsic mitochondrial respiration capacity of murine oxidative and glycolytic skeletal muscles, and its relationship with mitochondrial homeostasis and fiber composition, has not been comprehensively examined. We therefore evaluated mitochondria-specific aging by assessing the respiratory capacity of mitochondria isolated from muscles enriched in either fast-glycolytic or slow-oxidative fibers. We compared the tibialis anterior (a fast muscle mainly composed of type IIB and IIX fibers) and the soleus (a slow/fast muscle comprising type I, IIA and IIX fibers), which are representative of glycolytic and oxidative muscles, respectively [1,23,24]. We analyzed the respiration of isolated mitochondria because assessment of the mitochondrial respiratory capacity of permeabilized/ non-permeabilized muscle fibers and permeabilized muscles can be affected by substrate uptake or other active pathways related to fiber integrity and mitochondrial turnover. We also evaluated mitochondrial homeostasis pathways, including the expression of mitochondria-associated factors in the mitochondrial fraction, as well as muscle fiber composition, to provide a possible mechanistic explanation of the observed differences in mitochondrial respiration.

## RESULTS

### In young mice, the soleus is more oxidative than the tibialis anterior, and expresses higher levels of mitochondrial biogenesis, fission/fusion and autophagy markers

**Figure 1:**
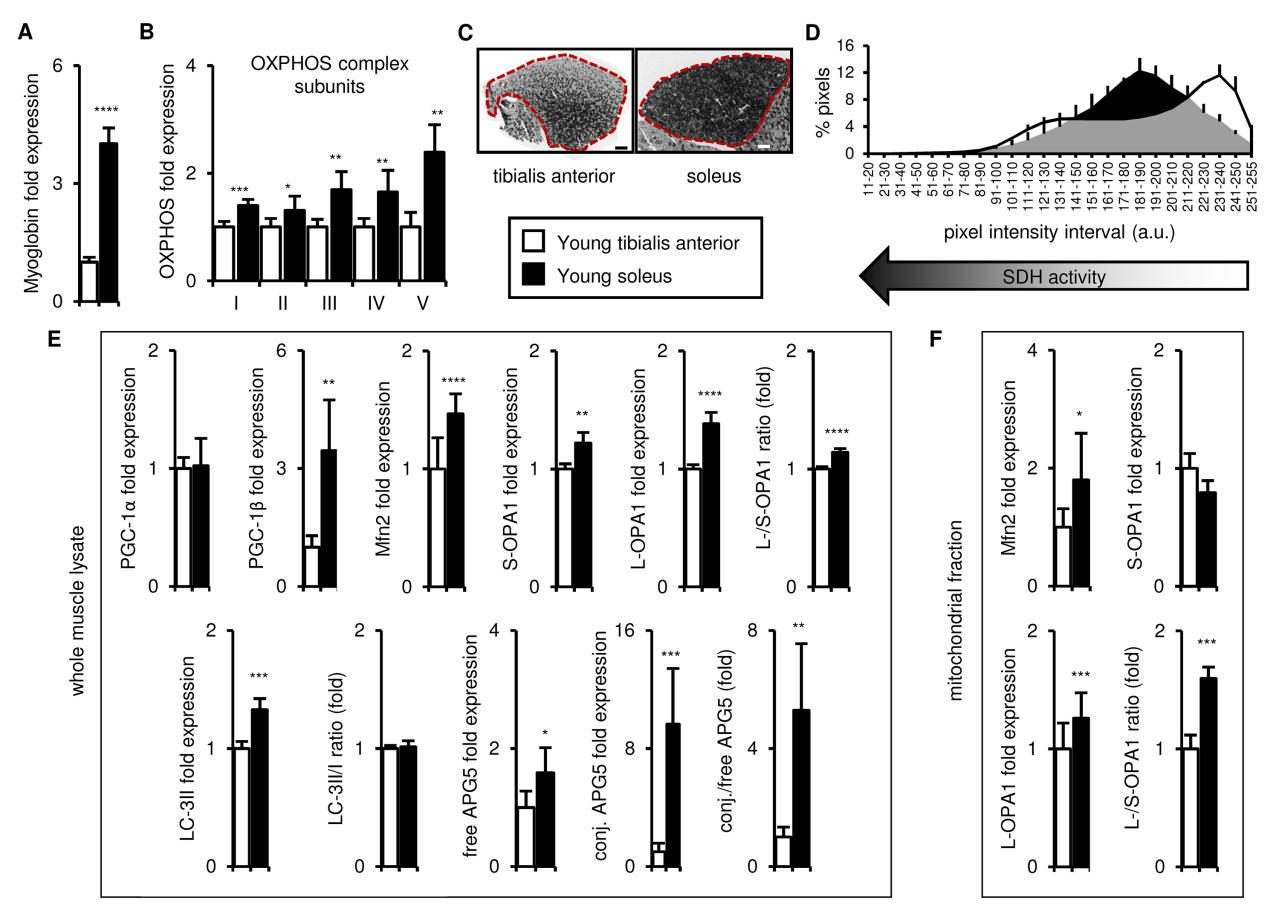
**In young mice, the soleus is more oxidative than the tibialis anterior, and expresses higher levels of mitochondrial biogenesis, fission/fusion and autophagy markers.** Markers of oxidative metabolism, mitochondrial biogenesis, fission/fusion and autophagy were evaluated in tibialis anterior and soleus muscles from young (3 mo) mice. (**A-B**) Myoglobin and representative electron transport chain enzymes (OXPHOS) in whole muscle lysates were assessed by Western blotting and normalized to Ponceau-stained total protein (see Figure S1). The mean plus standard deviation of 3 technical replicates of lysates from 5 mice/group is shown. (**C-D**) Succinate dehydrogenase (SDH i.e. OXPHOS Complex II) activity was assessed by histochemical staining. Representative images are shown (C; tibialis anterior scale bar = 400 µm, soleus scale bar = 200 µm) and the SDH activity of the entire muscle sections (indicated by the red dotted lines i.e. the EDL and gastrocnemius were excluded) was evaluated by assessing pixel intensities (D; mean plus standard deviation of 3 mice/group, 2 sections per mouse). (**E**) PGC-1α, PGC-1β, Mfn2, short (S)- and long (L)-OPA1, LC3-II/I and APG5 in whole muscle lysates were evaluated by Western blotting and normalized to Ponceau-stained total protein (see Figure S1). The mean plus standard deviation of 3 technical replicates of lysates from 5 mice/group is shown. (**F**) Mfn2 and OPA1 in mitochondrial fractions were evaluated by Western blotting and normalized to Ponceau-stained total protein (see Figure S1). The mean plus standard deviation of lysates from 4 mice/group is shown. *p ≤ 0.05, **p ≤ 0.01, ***p ≤ 0.001, ****p ≤ 0.0001.

We first compared the metabolic identity of the tibialis anterior and soleus from young (3 month old) mice. As expected, the tibialis anterior and soleus were metabolically distinct. The soleus was more oxidative than the tibialis anterior, as indicated by its higher expression of both myoglobin (an indirect indicator of oxidative phosphorylation [25];) and representative mitochondrial respiratory complex enzymes (OXPHOS complex subunits; (Figures 1A-B and S1A)). We also assessed the succinate dehydrogenase (SDH) activity of the entire muscle cross-sections. As expected, given the known fiber type complexity of the tibialis anterior, we observed a broad range of SDH activity, with areas of higher and lower SDH activity differentially distributed across the muscle section, which highlighted the importance of carefully collecting and analyzing the entire muscle (Figure 1C-D). In contrast, uniformly high SDH activity was observed in the soleus (Figure 1C-D), consistent with the presence of type I oxidative and type IIA-IIX oxidative/glycolytic fibers.

To determine whether the distinct metabolic nature of the two muscles corresponded to differences in mitochondrial homeostasis, we next evaluated the expression of markers of mitochondrial biogenesis (PGC-1α and PGC-1β [2,15,26]), fission/fusion (Mfn2, OPA1 [27,28]), and autophagy (LC3-II/I [29]; APG5 [30]). Levels of PGC-1β, Mfn2 and OPA1 (both short and long isoforms) were higher in whole muscle lysates of the soleus compared to the tibialis anterior, and the long/short OPA1 isoform ratio was also greater in the soleus (Figures 1E and S1A-C). Levels of LC3-II and both free and conjugated APG5, as well as the conjugated/free APG5 ratio, were also higher in the soleus, compared to the tibialis anterior (Figures 1E and S1A-B). No differences in PGC-1α levels or the LC3-II/I ratio were observed (Figures 1E and S1A-B). We also evaluated Mfn2 and OPA1 expression in mitochondrial fractions of the two muscles to verify that the differences don’t simply reflect the higher mitochondrial content of the soleus, and similarly observed more Mfn2 and a higher long/short-OPA1 isoform ratio in the soleus (Figures 1F and S1D). Thus, taken together, these data indicate that the soleus possesses more of the machinery for mitochondrial turnover and mitophagy than the tibialis anterior.

### In old mice, the soleus remains more oxidative than the tibialis anterior, and expresses higher levels of mitochondrial biogenesis, fission/fusion and autophagy markers

**Figure 2:**
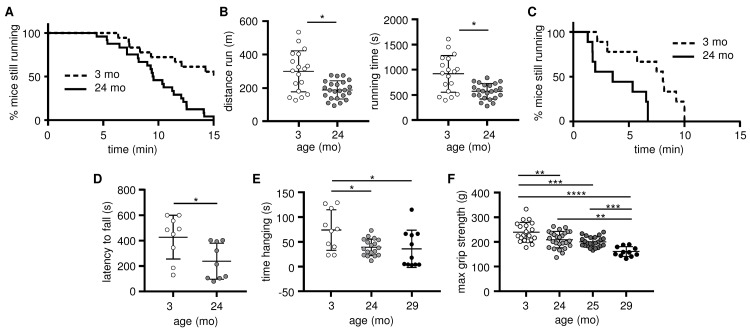
**Skeletal muscle strength and endurance, as well as overall exercise performance, decline as early as 24 months of age.** Exercise performance and skeletal muscle strength were evaluated in young (3 mo, open circles) and old (24-25 mo, grey circles; 29-30 mo, black circles) mice. (**A-B**) Exercise performance on a treadmill is presented as the percentage of mice still running (A) and the distance run and running time (B; means and standard deviations are shown); 3 mo (n=20), 24-25 mo (n=24). (**C-D**) Exercise performance on a rotarod is presented as the percentage of mice still running (C), and latency (D; mean time before falling and standard deviations are shown); 3 mo (n=9), 24 mo (n=9). (**E**) Muscle endurance assessed using a hang wire test is presented as time spent hanging on the wire before falling (means and standard deviations are shown); 3 mo (n=11), 24 mo (n=18), 29 mo (n=11). (**F**) Muscle strength was assessed using a four-limb grip strength test (means and standard deviations are shown); 3 mo (n=20), 24 mo (n=26), 25 mo (n=24), 29 mo (n=11). *p ≤ 0.05, **p ≤ 0.01, ***p ≤ 0.001, ****p ≤ 0.0001.

We next conducted a series of assessments of exercise performance and muscle strength to evaluate sarcopenia onset in our mouse colony, and select the age at which old mice are sarcopenic for subsequent comparisons with young mice. Performance in a treadmill test (Figure 2A-B) and on a rotarod (Figure 2C-D) was reduced by 24 months of age, although this may in part reflect increased body weight (Figure S2A-B) and/or a decline in cardiac function. 24-month-old mice also performed less well in a hang-wire test (Figure 2E), although this may similarly be partially attributed to their higher body weight (Figure S2C). To specifically evaluate skeletal muscle strength, we therefore used the four-limb grip strength test (Figure 2F), which is not affected by body weight (Figure S2D ) and observed reduced skeletal muscle strength by 24 months of age, with a further reduction by 29 months of age. For subsequent experiments, we therefore compared 3-month “young” mice with 28-29-month “old” mice.

**Figure 3:**
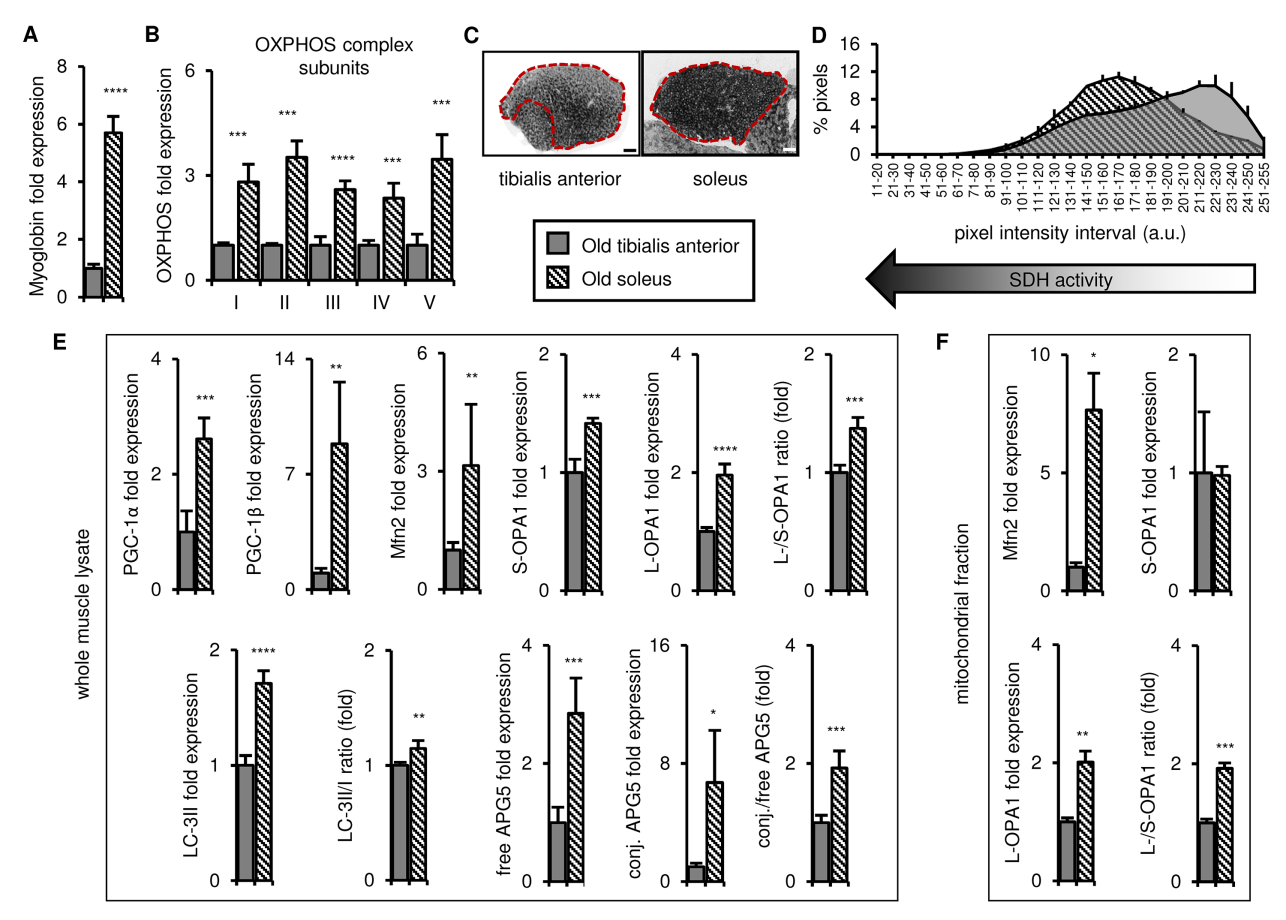
**In old mice, the soleus remains more oxidative than the tibialis anterior, and expresses higher levels of mitochondrial biogenesis, fission/fusion and autophagy markers.** Markers for oxidative metabolism, mitochondrial biogenesis, fission/fusion and autophagy were evaluated in tibialis anterior and soleus muscles from old mice (28-29 mo). (**A-B**) Myoglobin and representative electron transport chain enzymes (OXPHOS) in whole muscle lysates were assessed by Western blotting and normalized to Ponceau-stained total protein (see Figure S3). The mean plus standard deviation of 3 technical replicates of lysates from 4 mice/group is shown. (**C-D**) Succinate dehydrogenase (SDH i.e. OXPHOS Complex II) activity was assessed by histochemical staining. Representative images are shown (C; tibialis anterior scale bar = 400 µm, soleus scale bar = 200 µm) and the SDH activity of the entire muscle sections (indicated by the red dotted lines i.e. the EDL and gastrocnemius were excluded) was evaluated by assessing pixel intensities (D; mean plus standard deviation of 3 mice/group, 2 sections per mouse). (**E**) PGC-1α, PGC-1β, Mfn2, short (S)- and long (L)-OPA1, LC3-II/I and APG5 in whole muscle lysates were evaluated by Western blotting and normalized to Ponceau-stained total protein (see Figure S3). The mean plus standard deviation of 3 technical replicates of lysates from 4 mice/group is shown. (**F**) Mfn2 and OPA1 in mitochondrial fractions were evaluated by Western blotting and normalized to Ponceau-stained total protein (see Figure S3). The mean plus standard deviation of lysates from 4 mice/group is shown. *p ≤ 0.05, **p ≤ 0.01, ***p ≤ 0.001, ****p ≤ 0.0001.

We next compared the tibialis anterior and soleus muscles in old mice and found that the soleus remained more oxidative than the tibialis anterior, with higher expression of myoglobin and representative OXPHOS complex subunits (Figures 3A-B and S3A), and higher SDH activity (Figure 3C-D). The markers of mitochondrial biogenesis, dynamics and autophagy (PGC-1α/β, long OPA1, LC3-II, free and conjugated APG5, and the long/short OPA1, LC3-II/I, and conjugated/free APG5 ratios) were also higher in the whole muscle lysates of the old soleus than the old tibialis anterior (Figures 3E and S3A-B), and Mfn2 expression and the long/short OPA1 ratio were also higher in the soleus mitochondrial fraction (Figures 3F and S3C). Taken together, these data demonstrate that the differences observed between the tibialis anterior and the soleus in young mice are maintained with age, consistent with higher mitochondrial turnover and dynamics in the soleus throughout life, which may contribute to mitochondrial function maintenance during aging [14,21,31–34].

### Mitochondrial function declines in the tibialis anterior but is maintained in the soleus with aging

**Figure 4:**
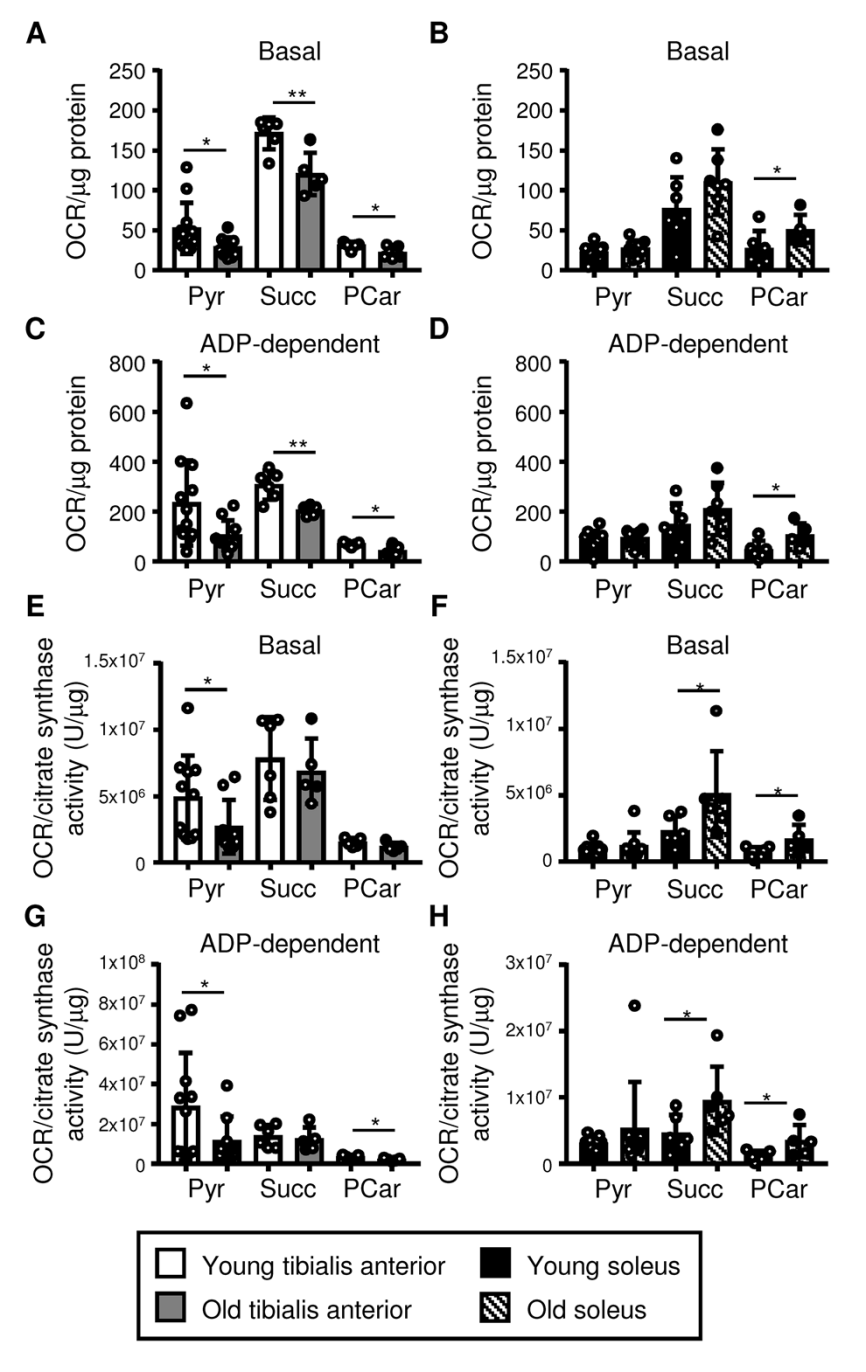
**Mitochondrial respiration declines in the tibialis anterior, but it is maintained in the soleus upon aging.** Respiration in mitochondrial fractions isolated from the tibialis anterior (**A, C, E, G**) and soleus (**B, D, F, H**) muscles of young (3 mo) and old (28-29 mo) mice was assessed using pyruvate (Pyr), succinate (Succ), and palmitoylcarnitine (PCar) as substrates (see also Figures S4 and S5). Basal and ADP-dependent mitochondrial respiration is shown. Data were normalized for either the protein content of the mitochondrial fraction (A-D; see Figure S4B) or citrate synthase activity/µg of protein (E-H; see Figure S4C), and the means plus standard deviations of 6-11 (Pyr) or 5-6 (Succ, PCar) mice/group are shown. Congenic CD45.2 and CD45.1 old mice were used (young mice were all CD45.2): white dots = CD45.2, black dots = CD45.1. *p ≤ 0.05, **p ≤ 0.01.

We next assessed the function of mitochondria isolated from the tibialis anterior and the soleus using Seahorse respirometry (as outlined in Figure S4A). Mitochondrial oxygen consumption was evaluated using pyruvate, succinate, and palmitoyl-carnitine as substrates, to assess Complex I-dependent respiration, Complex II-dependent respiration, and coordination between β-oxidation and the electron transport chain, respectively [35,36]. The choice of an appropriate normalization factor is crucial to obtain reliable results [3,10]. We therefore evaluated the protein content and citrate synthase activity of the mitochondrial fraction as potential normalization factors. We did not observe any differences between the young and old samples of each muscle type (Figure S4B-C), suggesting that normalization to either mitochondrial fraction protein content or citrate synthase activity would be reasonable. Normalizing mitochondrial respiration to protein content and citrate synthase activity yielded similar results, which are both shown for completeness (Figure 4A-D, normalized for protein content; Figure 4E-H, normalized for citrate synthase activity). Basal and ADP-dependent mitochondrial respiration declined upon aging in the tibialis anterior when pyruvate, succinate and palmitoyl-carnitine were used as substrates and the data were normalized for protein content (Figure 4A and C). When the data were normalized for citrate synthase activity, basal and ADP-dependent respiration with pyruvate similarly declined upon aging, and ADP-dependent respiration declined with palmitoyl-carnitine, while no differences between young and old mitochondria were observed with succinate (Figure 4E and G). In contrast, neither basal nor ADP-dependent respiration by the soleus mitochondria declined upon aging when either normalization method was used (Figure 4B, D, F, H). Indeed, basal and ADP-dependent respiration with succinate increased in mitochondria from the soleus when the data were normalized to citrate synthase activity (Figure 4F and H), and both normalization methods revealed higher basal and ADP-dependent respiration with palmitoyl-carnitine (Figure 4B, D, F and H).

**Figure 5:**
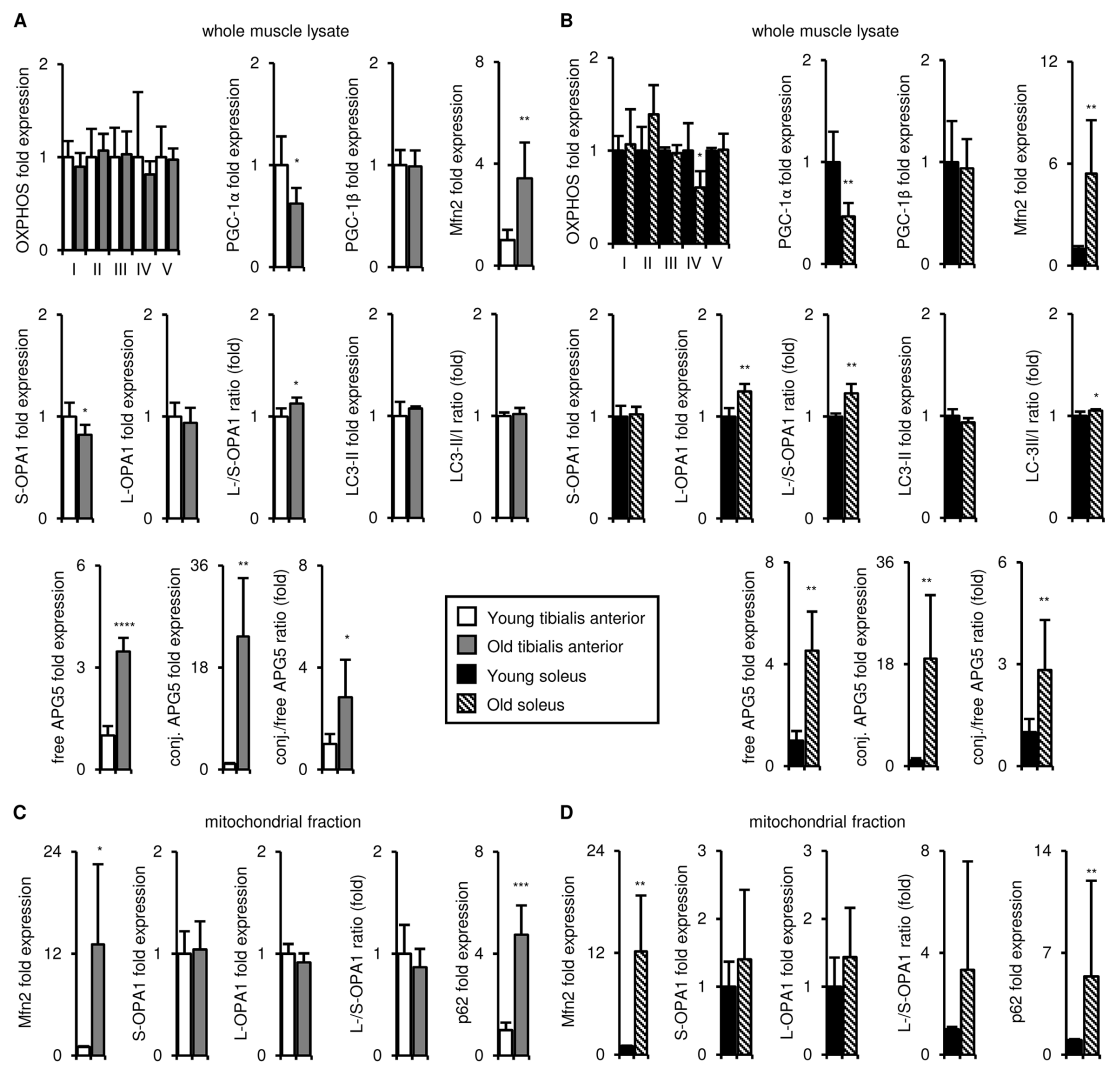
**Upon aging, the tibialis anterior and soleus undergo similar changes in mitochondrial biogenesis, fission/fusion, disposal and autophagy marker expression.** Markers of mitochondrial biogenesis, fission/fusion, disposal and autophagy were evaluated in tibialis anterior (**A, C**) and soleus (**B, D**) muscles isolated from young (3 mo) and old (29 mo) mice. A-B) Representative OXPHOS subunits, PGC-1α, PGC-1β, Mfn2, short (S)- and long (L)-OPA1, LC3-II/I, and APG5 in whole muscle lysates were evaluated by Western blotting and normalized to Ponceau-stained total protein (see Figures S6, S7). The mean plus standard deviation of 3 technical replicates of lysates from 4-5 mice/group is shown; 3 mo (n=5), 29 mo (n=4). C-D) Mfn2, OPA1, and p62 levels in mitochondrial fractions were evaluated by Western blotting and normalized to Ponceau-stained total protein (see Figure S7). The mean plus standard deviation of lysates from 4 mice/group run on 2 separate gels is shown. *p ≤ 0.05, **p ≤ 0.01, ***p ≤ 0.001, ****p ≤ 0.0001.

Respirometry analysis of isolated mitochondria has previously been questioned because mechanical stress during mitochondrial isolation may lead to uncoupling between respiration and ATP production [5]. However, our optimized experimental design resulted in consistent mitochondrial responses among independent technical and biological replicates, and the Respiratory Control Index (RCI; an indicator of the tightness of coupling between respiration and phosphorylation) was not different between young and old muscles (Figures S4 and S5). Thus, the observed differences do not appear to be an artifact of tissue homogenization. The lack of differences in the RCI between young and old muscles also indicates that the tightness of coupling between respiration and phosphorylation does not change upon aging in either the tibialis anterior or the soleus. Moreover, the observed changes in mitochondrial respiration were not due to differences in the amount of mitochondria because the data were normalized, and in any case, the citrate synthase activity within the mitochondrial fraction was similar in the young and old muscles (Figure S4C), as were the mitochondrial fraction mass (Figure S4B) and levels of the representative OXPHOS complex subunits in the whole muscle lysates (Figure 5A, S6A-B). Surprisingly, we did not observe any differences in mtDNA integrity (single or double-strand DNA breaks or bulky adducts, detected by long-range PCR) between young and old tibialis anterior and soleus (Figure S5G), although we did not perform mtDNA sequencing to detect point mutations or deletions.

### The tibialis anterior and soleus undergo similar aging-associated changes in mitochondrial biogenesis, fission/fusion, disposal and autophagy marker expression

The literature on aging-associated changes in mitochondrial homeostasis in the skeletal muscle is controversial (reviewed in [6]). In our studies, markers of mitochondrial homeostasis were more highly expressed in the soleus than in the tibialis anterior at 3 months of age (Figures 1 and S1). We hypothesized that preservation or further exacerbation of those differences upon aging might underlie the maintenance of mitochondrial function in the soleus, but not the tibialis anterior. To test this, we evaluated the mitochondrial properties of these muscles in young and old mice. As noted above, the mass of the mitochondrial fraction did not change upon aging for either muscle (Figure S4B). Levels of the OXPHOS complex subunits also remained mostly constant in both muscles, apart from Complex IV (cytochrome c oxidase), which declined upon aging in the soleus (Figures 5A-B and Figure S6A-B). PGC-1α levels decreased upon aging in both muscles, whereas PGC-1β expression was maintained (Figures 5A-B and Figure S6C-F). Mfn2 levels increased in the whole muscle lysates and the mitochondrial fractions of both muscles (Figures 5 and Figure S7A-B), but OPA1 expression was not notably altered upon aging in either the whole muscle lysates or the mitochondrial fractions (Figures 5 and Figure S6A-B). The LC3-II/I ratio did not change notably in either muscle (Figures 5A-B and Figure S7C-D), but free and conjugated APG5 levels, as well as the conjugated/free APG5 ratio, increased with age in both muscles (Figures 5A-B and Figure S6C-D). Levels of p62, a marker of mitochondrial targeting for disposal, increased in the mitochondrial fraction of both muscles (Figures 5A-B and Figure S7A-B). Taken together, although we observed aging-associated changes in mitochondrial markers, these changes were similar in the tibialis anterior and the soleus, and could not therefore explain the observed aging-associated differences in mitochondrial respiration.

### The tibialis anterior and soleus exhibit distinct changes in muscle fiber composition upon aging

**Figure 6:**
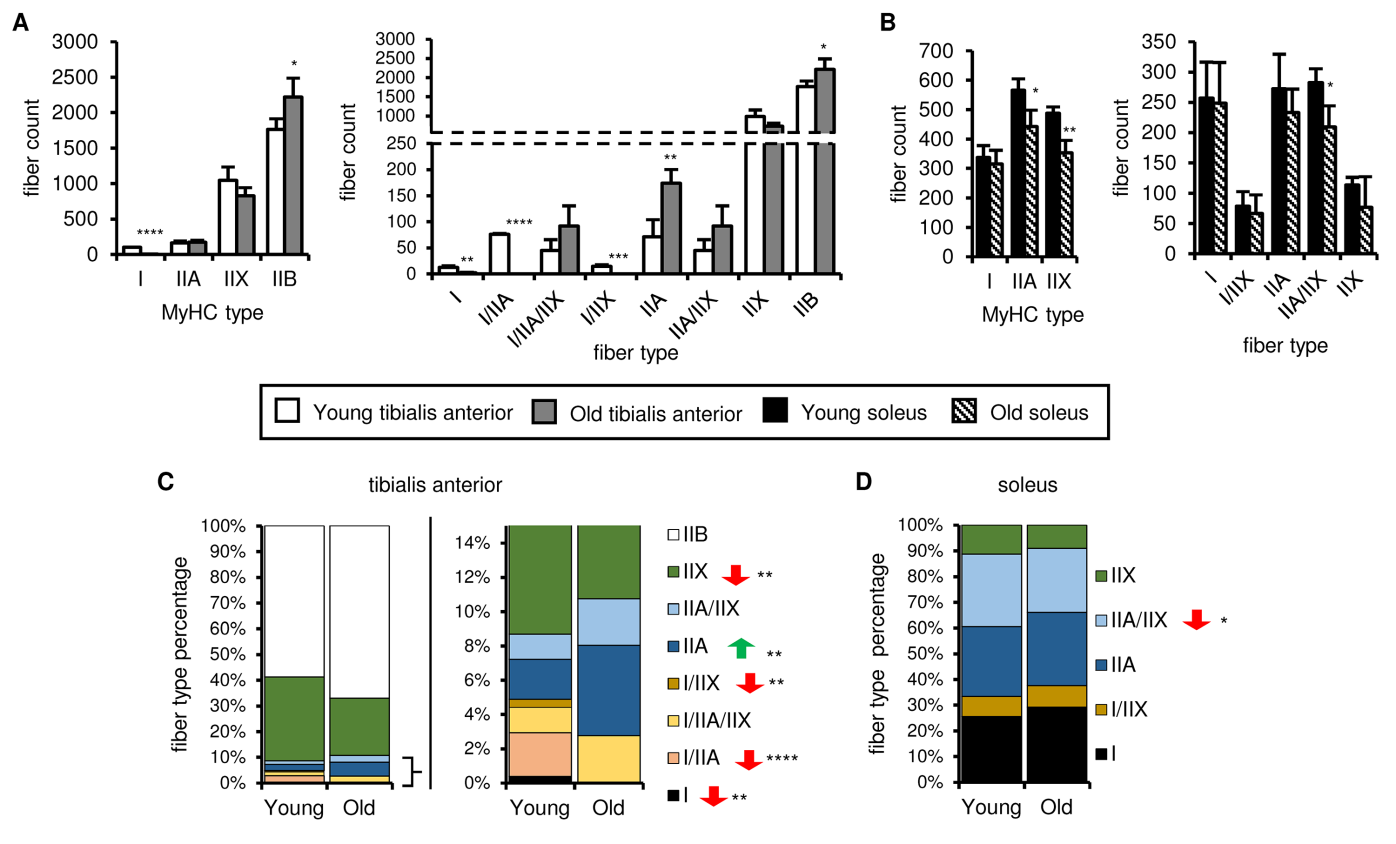
**Fiber type number and muscle fiber composition show different age-related changes in the soleus and tibialis anterior.** The myosin heavy chain (MyHC) fiber composition of tibialis anterior (**A, C**) and soleus (**B, D**) muscles from young (3 mo) and old (28-29 mo) mice was assessed by immunostaining (see also Figure S8). Total type I, IIA, IIX and IIB MyHC-expressing fiber counts (A-B, left panels), counts for each fiber type (based on single, double or triple positivity for type I, IIA, IIX and IIB MyHC; (A-B, right panels), and proportions of each fiber type (C-D) are shown. All data are means plus standard deviations of 2 sections/mouse, 3 mice/group. Fiber types are arranged in approximate order of most oxidative (type I) to most glycolytic (type IIB). *p ≤ 0.05, **p ≤ 0.01, ***p ≤ 0.001, ****p ≤ 0.0001.

An alternative explanation is that aging-associated changes in muscle fiber composition underlie the differences in mitochondrial respiration, because mitochondrial quality and homeostasis are fiber type-specific. We therefore evaluated the fiber composition of young and old tibialis anterior and soleus muscles using antibodies specific for the isoforms of myosin heavy chain (MyHC) that characterize slow-oxidative (type I) and fast-oxidative/glycolytic (types IIA, IIX, IIB) fibers. Representative images are shown in Figure S8. Type I-MyHC-expressing fibers were present in the tibialis anterior muscles from young mice, albeit in small numbers, and often completely absent in old tibialis anterior muscles (Figure 6A). The number of type IIA-MyHC-expressing fibers was similar in young and old tibialis muscles, whereas type IIB-MyHC-expressing fiber counts increased with age (Figure 6A). Type I-MyHC-expressing fiber counts were not affected by aging, whereas type IIA-MyHC- and type IIX-MyHC-expressing fiber counts decreased upon aging (Figure 6B). However, many fibers expressed more than one MyHC isoform, especially in the tibialis anterior. We therefore defined each fiber as either expressing a single MyHC isoform or a mixture of isoforms. In the tibialis anterior, the number and percentage of type I, I/IIA, IIA and I/IIX fibers decreased upon aging, whereas the number of type IIB fibers increased (Figure 6A and C), resulting in an enrichment of more glycolytic fiber types and a decreased complexity, consistent with the aging-associated changes in SDH activity observed on the whole muscle sections (Figures 1C-D and 3C-D). In the soleus, the number and percentage of type IIA/IIX fibers decreased upon aging, but no other changes in the fiber types were observed (Figure 6B and D), indicating that the oxidative-glycolytic fiber balance of the soleus was maintained upon aging. Taken together, these data suggest that muscle-specific changes in fiber composition may contribute to the overall aging-associated effects on mitochondrial respiration.

## DISCUSSION

Mitochondrial respiration is of fundamental importance to ensure efficient ATP production, avoid oxidative stress and maintain cell viability. Skeletal muscle relies on efficient mitochondrial function for myofilament contraction as well as cellular homeostasis. Previous studies reported aging-associated mitochondrial function decline in the fast-glycolytic muscles of rats and their isolated fibers [3–5,10], in permeabilized murine muscles [7], and in mitochondria isolated from the fast-glycolytic/oxidative muscles of human subjects [8], while results in oxidative muscles are somewhat controversial [3,5,7,37]. Similarly, the literature on mitochondrial homeostasis is controversial (reviewed in [6]). In this study, we therefore evaluated aging-associated changes in mitochondrial function, mitochondrial homeostasis and fiber composition in murine muscles with different contractile functions and metabolic needs by comparing the tibialis anterior, which is strongly enriched in type IIB (fast glycolytic) and IIX (fast oxidative/glycolytic) fibers, and the soleus, which consists of a mixture of type I (slow oxidative) and IIA/IIX (fast oxidative/glycolytic) fibers.

Our data demonstrate that mitochondrial respiratory capacity does not decline homogeneously in oxidative and glycolytic muscles. Indeed, along with previous studies using different approaches in rats and mice, our data demonstrate that mitochondrial function only declines in fast-glycolytic muscles upon aging [3,7,10]. Higher oxidative phosphorylation corresponds to greater expression of the machinery for mitochondrial homeostasis and autophagy, and sustained higher expression of those factors throughout life, which may protect soleus mitochondria from functional deterioration. Indeed, increased expression of PGC-1α and PGC-1β was previously shown to improve skeletal muscle health [19,38–41]. Moreover, autophagy plays a fundamental role in mitochondrial homeostasis, and skeletal muscle development and function [20], and inhibition of autophagy induced mitochondrial abnormalities and muscle weakness in adult mice [31,32], although the role of autophagy in sarcopenia remains controversial [32]. Therefore, lifelong higher expression of PGC-1α/β and autophagy factors may suggest better mitochondrial homeostasis, resulting in overall preserved muscle function upon aging.

Mfn2 and OPA1 play fundamental roles in mitochondrial fusion and fission, which are crucially important mechanisms for mitochondrial quality control that allow segregation of damaged mitochondrial components, and their elimination by autophagy [27,28], thereby maintaining mtDNA stability and tolerance of mtDNA mutations [33]. The higher expression of Mfn2 and OPA1 observed in the soleus is particularly interesting because two recent studies showed that Mfn2 and OPA1 deficiencies are strongly involved in sarcopenia, aging-associated oxidative stress and unfolded protein response activation [14,34]. We did not detect differences in mtDNA single- and double-strand breaks using a long-range PCR amplification assay.

Since oxidative metabolism seems to drive mitochondrial homeostasis [12,17,19–21,31–33], the relatively higher oxidative metabolism should be linked to better mitochondrial homeostasis at the fiber level. Therefore, the capacity of muscle fibers to maintain mitochondrial function with age should be highest in type I oxidative fibers, and progressively decline in the more glycolytic fiber types. Consistent with that, the decreased abundance of oxidative fibers and increase in glycolytic fibers observed in the tibialis anterior with aging, may contribute to the overall effect on mitochondrial respiration. On the other hand, in the soleus, the fibers with better mitochondrial homeostatic mechanisms are maintained with aging, whereas the fibers with a lower oxidative metabolism, and likely less efficient mitochondrial turnover and function, decrease. This may contribute to the maintenance of mitochondrial respiration in the soleus. Interestingly, a recent study confirmed the fiber specificity of mitochondrial homeostasis in the context of skeletal muscle aging in humans [42].

We focused on the tibialis anterior and soleus as representative large muscles important for mobility in both mice and humans. These opposing muscles control foot dorsiflexion and plantarflexion, respectively. Both are essential for walking and balance. Muscle functional defects in disease and aging are influenced by muscle location and contractile properties as well as metabolic effects [43]. For instance, in Duchenne muscular dystrophy, contraction-induced injuries predominantly affect the calf and respiratory musculature (e.g. soleus, gastrocnemius and diaphragm), which undergo constant contraction to maintain posture and support breathing [44–47]. In metabolic diseases involving insulin modulation on the other hand, muscles are differentially affected depending on their relative abundance of glycolytic and oxidative fibers [48–50]. Apoptosis of individual myocytes, atrophy (including ubiquitin-proteasome degradation), MyHC isoform switch, fiber-specific responses [49] or a failure of satellite cells to replace certain subtypes of myocytes during normal cellular turnover [51] may also underlie loss of glycolytic muscle mass. Moreover, previous work has shown that sarcopenia is associated with downregulation of the atrogin-1 pathway [52] and thus appears to differ from denervation-induced atrophy, but may resemble skeletal muscle atrophy associated with heart failure [53]. The atrogin-1 pathway may therefore be an interesting target for future studies.

Physical exercise is currently recognized as the most effective strategy to prevent sarcopenia and related comorbidities, such as cognitive decline, metabolic diseases, osteoporosis, and frailty [54–58]. The function of skeletal muscle, the body’s biggest “organ”, is a fundamental piece in the complex puzzle of body aging. Indeed, a recent study demonstrated that deletion of OPA1 in skeletal muscle recapitulated aging-associated features in other organs (such as disruption of metabolic homeostasis, and induction of epithelial senescence and systemic inflammation), leading to mouse death in ~100 days [34].

Our findings suggest that in order to improve overall mitochondrial quality, and prevent functional decline, sarcopenia and related comorbidities, exercise designed to enrich the skeletal muscles in oxidative fibers (e.g. by endurance or weight training) may represent a more valuable approach than non-specific physical exercise. Indeed, although aging-associated development of skeletal muscle atrophy seems to be independent of some features of mitochondrial aging [10,59,60], it is worth noting that in recent studies, aerobic exercise improved muscle strength and endurance in human subjects, without increasing muscle mass [61]. This suggests that improving oxidative metabolism may positively affect skeletal muscle function in different ways, such as improving mitophagy and mitochondrial homeostasis [59]. In this context, our study provides possible mechanistic explanations for recent findings related to the anti-aging effects of physical exercise on skeletal muscle [61–63] and further reinforces the idea that exercise training aimed at improving oxidative metabolism may be the most complete and direct approach to prevent mitochondria-dependent skeletal muscle aging, and to delay the onset of sarcopenia-related diseases [64–69].

Finally, the function of satellite cells, which can either form new fibers or fuse with pre-existing fibers, declines with age, thereby contributing to the establishment and progression of sarcopenia due to their decreased capacity to replace degenerating fibers. Satellite cells, which are located underneath the basal lamina of each muscle fiber, have distinct features when associated with fast or slow fibers, and also exhibit a pre-determined fate towards a more slow or fast phenotype [70]. Oxidative stress and mitochondrial aging are fundamental mechanisms in satellite cell aging [71–73]. Satellite cells associated with oxidative fibers may have better mitochondrial homeostasis and a greater capacity to maintain mitochondrial function with age, thereby contributing to the aging-associated differences between oxidative and glycolytic muscles.

Future studies using single-cell analysis approaches [42] coupled with functional analysis of mitochondrial respiration and non-invasive imaging [74] to evaluate the impact of oxidative metabolism on mitochondrial homeostasis, fiber contractile machinery, and cellular pathways, may lead to the identification of novel targets for anti-aging therapies.

## MATERIALS AND METHODS

### Animals

Male C57BL/6J mice were purchased from The Jackson Laboratory (Bar Harbor, ME) at 3 months of age (young mice) or 12-18 months of age (old mice, which were further aged in house), and maintained in an SPF facility at Cedars-Sinai Medical Center. All procedures were performed with Institutional Animal Care and Use Committee approval. Some congenic CD45.1-expressing (PepBoy) mice were used in addition to regular CD45.2-expressing C57BL/6J mice for mitochondrial respiration analyses, as indicated in the Figure Legends. Mitochondrial homeostasis is very sensitive to circadian and fasting/feeding cycles, CO_2_/O_2_ balance, and post-mortem and *ex vivo* modifications, so we optimized euthanasia and sample harvesting procedures to minimize bias.

### Exercise performance assessment

All exercise performance tests were performed by the same skilled evaluator, and who was blinded to the mouse groups. Mice were acclimated to the room for 10-15 min before each session.

*Treadmill test:* Running endurance was assessed using a 10˚-inclined Exer 3/6 Treadmill (Columbus Instruments, Columbus, OH) and Romatex Treadmill Software v3.2.8. Mice were acclimated to the treadmill 2 days before performance assessment (5 min at a speed of 6 m/min with repetitive taps on the back anytime they stopped running, followed by 5 min at 11 m/min with a small shock applied to the grid to motivate running). On the day of assessment, mice ran a 5 min warmup (a gradual increase in speed from 8 m/min to 10 m/min over 1 min, followed by 4 min at a constant speed of 10 m/min). Immediately after the warmup, the treadmill speed was increased with a constant acceleration, and running performance was assessed over a 15 min interval when the speed increased from 10 to 20 m/min over the first 10 min, and then remained constant at 20 m/min for the last 5 min. The length of time the mice ran before exhaustion (defined as refusal to run after repetitive taps on the back) was recorded (excluding the warmup phase).

*Rotarod test:* Mice were acclimated to the 6.98 cm diameter rotarod (San Diego Instruments, San Diego, CA) on 2 consecutive days (2 rpm for 5 min each day) to train the mice not to jump from the rotor, prior to the assessment of rotarod performance. The test day consisted of a warmup phase (2 rpm for 3 min) and an orientation phase (1 min without rotation to orient the mice), prior to a 10 min evaluation phase during which the rotarod speed increased from 3 to 10 rpm. The length of time the mice remained on the rotor before falling (latency) was recorded (excluding the warmup and orientation phases).

*Four-limb hang-wire test:* Mice were placed on an iron grid (grid squares size: ~ 6x6 mm). The grid was shaken to ensure the mice gripped tightly with all four paws, and the grid was then inverted over a Plexiglas cylinder ~ 35 cm above a foam pad. Hang-wire performance was evaluated by assessing the length of time the mouse remained attached to the grid before falling.

*Four-limb grip strength test:* Four-limb grip strength was evaluated using an Animal Grip Strength System (San Diego Instruments, San Diego, CA) by allowing the mouse to grab a flat horizontal grid with all four limbs, and then pulling the mouse away from the grid by the tail in a horizontal movement. The force exerted by the mouse grabbing the grid was measured. Mice were assessed three consecutive times, and the maximum force measured during the three assessments was noted.

### Evaluation of protein levels in whole muscle lysates and mitochondrial fractions

Mitochondrial homeostasis is very sensitive to circadian and fasting/feeding cycles, CO_2_/O_2_ balance, and post-mortem and ex vivo modifications, so we optimized euthanasia and sample harvesting procedures to minimize bias.

*Muscle isolation:* To study key regulators of mitophagy and biogenesis, mice were anesthetized (100 mg/Kg ketamine + 10 mg/Kg xylazine i.p.) and then euthanized by cervical dislocation to avoid exposure to CO_2_, and specific individual muscles were carefully isolated within 2-3 min of euthanasia. The epimysium was removed, and the tissue was quickly frozen in liquid nitrogen or immediately homogenized for mitochondrial isolation.

*Preparation of whole tissue lysates:* Protein extracts were isolated using RIPA buffer (50 mM Tris-HCl pH 7.4, 1% NP-40, 0.5% Na-deoxycholate, 0.1% SDS, 150 mM NaCl, 2 mM EDTA) supplemented with 100x P8340 Protease inhibitor cocktail, and phosphatase inhibitors (NaF 50mM; Sigma-Aldrich, Carlsbad, CA). Briefly, tissue was homogenized in cold RIPA buffer using a pre-cooled Polytron homogenizer (PowerGen 125, Thermo Fisher Scientific, Waltham, MA; 3 pulses of 5 sec each at max speed). Samples were left on ice for 10 min and then centrifuged at 13,000g for 10 min at 4˚C in a pre-cooled centrifuge. Samples were then quantified using a DC protein assay (Bio-Rad, Hercules, CA) and stored at -80˚C.

*Preparation of mitochondrial fraction lysates:* Muscles were quickly isolated from the animal and homogenized in 500 µl of HES buffer + 0.5% free fatty acids-free BSA (HES buffer: 70 mM sucrose, 210 mM mannitol, 5 mM HEPES, 1 mM EGTA, pH 7.4; Sigma-Aldrich, Carlsbad, CA) using a Polytron homogenizer (PowerGen 125, Fisher Scientific, Waltham, MA). Homogenized samples were centrifuged at 1000 g x 5 min at 4˚C in a precooled centrifuge, and the supernatant was then transferred to a new 1.5 ml conical tube and centrifuged at 7000 g x 15 min at 4˚C in a precooled centrifuge. The supernatant was discarded and the pellet was washed once with HES 0.5% BSA, and once with HES without BSA, at 7000 g x 15 min at 4˚C in a precooled centrifuge. Protein extracts were isolated from the pellet using RIPA buffer (50 mM Tris-HCl pH 7.4, 1% NP-40, 0.5% Na-deoxycholate, 0.1% SDS, 150 mM NaCl, 2 mM EDTA) supplemented with 100x P8340 Protease inhibitor cocktail, and phosphatase inhibitors (NaF 50mM) (Sigma-Aldrich, Carlsbad, CA). Samples were left on ice for 10 min and then centrifuged at 13,000g for 10 min at 4˚C in a pre-cooled centrifuge. Samples were then quantified using a DC protein assay (Bio-Rad, Hercules, CA) and frozen at -80˚C before use to facilitate complete lysis. Samples were stored at -80˚C.

*Electrophoresis and Blotting:* Samples were diluted in NuPAGE LDS Sample buffer (Invitrogen, Thermo Fisher Scientific, Waltham, MA) and boiled at 100˚C for 10 min. Samples were separated in 4-12% Bis-Tris gels (NuPage, Life Technologies, Camarillo, CA) and transferred on to PVDF membranes (Immobilon-FL, Millipore Sigma, Burlington, MA) using the NuPAGE-NOVEX system (Thermo Fisher Scientific, Waltham, MA). Appropriate protein transfer was assessed by membrane staining with Ponceau Red (Sigma-Aldrich, Carlsbad, CA), which was also used for normalization. Membranes were blocked in TBS + 1% TWEEN-20 + 5% Milk for 1 hour. Primary antibodies were incubated overnight at the following dilutions/concentrations: anti-mouse total OXPHOS rodent WB Antibody (EPR12370, Abcam, Burlingame, CA; 1:1000 (1.5 nM)), anti-mouse Myoglobin (A-9) (sc-74525, Santa Cruz Biotechnology, Santa Cruz, CA; 1:1000 (0.2 nM)), anti-mouse OPA1 (BD Transduction Laboratories^TM^, La Jolla, CA; 1:500 (0.5 nM)), anti-mouse PGC-1α (Abcam, Burlingame, CA; 1:1000 (0.1 nM)), anti-mouse PGC-1β (Abcam, Burlingame, CA; 1:500 0.2 nM), Mfn2 (sc-100560, Santa Cruz Biotechnology, Santa Cruz, CA; 1:500 (0.2 nM)), LC3A/B (Cell Signaling, Danvers, MA; 1:1000), APG5 (C-1, sc-133158, Santa Cruz Biotechnology, Santa Cruz, CA; 1:1000 (0.2 nM)), p62 (BD Transduction Laboratories^TM^; 1:500 (0.5 nM)). Secondary antibodies were: Goat anti-Rabbit IgG IRDye 800CW, IRDye® 680RD Goat anti-Mouse IgG (H+L) (Li-Cor Inc., Lincoln, NE). Membrane staining was visualized using an Odyssey CLx (Li-Cor Inc., Lincoln, NE) and analyzed using Image Studio 4.0 and Excel. Protein expression (immunoblots) was normalized to total protein content (Ponceau staining) using ImageJ as follows: the Ponceau Red-stained membrane was scanned and the image was transformed into 8-bit, the background was subtracted and the mean intensity of each lane was measured. Inverted values were calculated and used for normalization. A Prism Ultra Protein Ladder (10-245 kDa) (Abcam, Burlingame, CA) was used for assessment of band size, and blots were stripped with New Blot™ PVDF Stripping Buffer 5X (Li-Cor Inc., Lincoln, NE) as necessary.

### Mitochondrial isolation and functional assessment

Mitochondrial function is very sensitive to circadian and fasting/feeding cycles, CO_2_/O_2_ balance, and post-mortem and ex vivo modifications, so we optimized euthanasia and sample harvesting procedures to minimize bias.

Every experiment included at least 1 young and 1 old mouse, analyzed on the same Seahorse plate, using a XFe96 analyzer. Mice were i.p. injected with anesthetics (ketamine 100 mg/Kg + xylazine 10 mg/Kg) and euthanized by cervical dislocation, to avoid exposure to CO_2_. Muscles were quickly isolated and the mitochondrial fraction was isolated as described above, quantified using a DC protein assay (Bio-Rad, Hercules, CA), and diluted to appropriate concentrations. 25 µl mitochondrial isolate was loaded in each well of a 96-well Seahorse respirometry plate. Mitochondrial protein amounts, and the experimental set up, were optimized for each condition in pilot experiments and used for subsequent studies as follows: Tibialis - 1 µg/well for succinate/rotenone, 2 µg/well for pyruvate/malate, 4 µg for palmitoylcarnitine/malate; Soleus - 2 µg/well for all the substrates analyzed (pyruvate/malate, succinate/ rotenone, palmitoylcarnitine/malate). The plate containing the mitochondria was centrifuged at 2000 g x 20 min at 4˚C in a pre-cooled centrifuge. Substrate solutions were prepared in MAS buffer (70 mM sucrose, 220 mM mannitol, 5 mM KH_2_PO_4_, 5 mM MgCl_2_, 2 mM HEPES, 1 mM EGTA, 0.2% fatty-acid-free BSA, pH 7.4; Sigma-Aldrich, Carlsbad, CA, USA), pre-warmed to 37˚C, and gently added to the corresponding wells immediately before performing the respirometry assay (to avoid undesired uncoupling effects observed following prolonged pre-incubation with the substrate solution). 155 µl substrate solution was added to each well, for a final volume of 180 µl/well. The substrate final concentration in the well, designed based on previously described protocols [35,75,76] and on results from pilot experiments, was as follows: pyruvate/malate 5.5 mM/2.5 mM, succinate/ rotenone 5 mM/3 µM, palmitoylcarnitine/malate 55 µM/2.5 mM. The plate was then incubated in a non-CO_2_ incubator for 2 min at 37˚C. ADP (Sigma-Aldrich, Carlsbad, CA), oligomycin (Cayman Chemical, Ann Arbor, MI), FCCP (Cayman Chemical, Ann Arbor, MI) and Antimycin A (Cayman Chemical, Ann Arbor, MI) were added sequentially, and one measurement was taken after each injection. Before ADP injection, basal respiration was measured 3 times. The final concentrations of the drugs at the corresponding time of respirometry measurement was as follows: 0.2 mM ADP, 2 µM oligomycin, 0.9 µM antimycin A, and (i) 0.5 µM FCCP for the soleus with pyruvate/malate and palmitoylcarnitine/malate, (ii) 1.5 µM FCCP for the tibialis and the soleus with succinate/rotenone, and for the soleus with palmitoyl-carnitine/malate, and (iii) 2 µM FCCP for the tibialis with pyruvate/malate. Pyruvate, malate, succinate, rotenone, and palmitoylcarnitine were from Sigma-Aldrich, Carlsbad, CA. XFe96 sensor cartridges were hydrated overnight following the manufacturer’s instructions. Oxygen consumption rate (OCR) was measured using an XF^e^96 Seahorse Instrument (Agilent, Santa Clara, CA). For normalization, mitochondrial fraction protein amount was quantified using a DC protein assay (Bio-Rad, Hercules, CA), and citrate synthase activity in the mitochondrial fraction was quantified using a Citrate Synthase Activity Colorimetric Assay Kit (BioVision, Milpitas, CA), according to the manufacturers’ instructions.

### Histology

*Sample preparation:* Freshly isolated skeletal muscles were embedded in a thin layer of Tissue-Tek® O.C.T. Compound (Sakura® Finetek, VWR, TX) and frozen using isopentane (2-methylbutane; Sigma-Aldrich, Carlsbad, CA) cooled with liquid nitrogen to preserve optimal skeletal muscle morphology, as previously described [77,78]. Samples were completely embedded in OCT using ICE-IT^TM^ (Thermo Fisher Scientific, Waltham, MA) to avoid sample thawing, and 10 µm thick cryosections were obtained using a Leica CM3050 S Cryotome (Leica Microsystems Inc., Buffalo Grove, IL) at -22 ˚C. Sections were stored at -80˚C until needed.

*Fiber type staining and counting:* A PAP pen was used to create a hydrophobic border around the sections prior to permeabilization using cold methanol and incubation for 5 min at -20^o^C. After washing with PBS, endogenous IgG was blocked using Vector Mouse-On-Mouse (M.O.M; 2 drops in 2.5 ml PBS) for 30 min at room temperature (RT) in a humidified chamber (Vector Laboratories, Inc., Burlingame, CA). A subsequent blocking step was carried out using blocking buffer (5% horse serum (SH30074.03, GE Healthcare Biosciences Corp) + 0.1% Triton-X100 (Sigma-Aldrich, Carlsbad, CA) + 1% BSA (Sigma-Aldrich, Carlsbad, CA) in PBS (Mediatec Inc, Manassas, VA)) for 1 hour at RT in a humidified chamber. Serial slides were stained with anti-mouse laminin (Vector Laboratories, Inc., Burlingame, CA; 1:500 (1.9 mg/L)) and one of the myosin isoform antibodies: (i) anti-mouse type I (slow) myosin heavy chain (Abcam, Burlingame, CA, NOQ7.5.4D; 1:200), (ii) anti-mouse type IIA myosin heavy chain (SC-71; developed by S. Schiaffino, University of Padua for the NICHD Developmental Studies Hybridoma Bank, Iowa City, IA; 1:100), (iii) anti-mouse type IIB myosin heavy chain (BF-F3; developed by S. Schiaffino, University of Padua for the NICHD Developmental Studies Hybridoma Bank, Iowa City, IA; 1:70), (iv) anti-mouse type IIX myosin heavy chain (SH1-s; developed by S. Schiaffino, University of Padua for the NICHD Developmental Studies Hybridoma Bank, Iowa City, IA; 1:5). Secondary anti-rabbit IgG-Alexa Fluor 647, anti-mouse IgG-Alexa Fluor 594, and goat anti-mouse IgG-rhodamine antibodies (Thermo Fisher Scientific, Waltham, MA) were used according to the manufacturer’s instructions. Each primary antibody was diluted in blocking buffer and incubated in a humidified chamber for 2 hours or overnight. Each antibody incubation was followed by 2 washes with PBS + 1% BSA. Slides were then mounted using fluorescent mounting media (DAKO, Agilent, Santa Clara, CA). Samples to be compared were stained in the same staining session and using the same conditions. Images were either acquired using a Leica DM6000 B Fluorescent Microscope with LAS X v.2 software (Leica Microsystems Inc., Buffalo Grove, IL), or the entire slide was scanned using a TissueFAXS 200 with TissueFAXS Viewer 1016 software (TissueGnostics, Tarzana, CA). Positive fibers were counted on the section using ImageJ by an operator blinded to the age of the mouse.

*Succinate Dehydrogenase (SDH) activity:* SDH activity was assayed on tissue sections as previously described [79] using a staining protocol from the University of San Diego website (http://muscle.ucsd.edu/musintro/histochem.shtml), by incubating the slides at 37˚C for 30 min in 0.05 M phosphate buffer pH 7.6 containing 1.53 mM nitroblue tetrazolium and 0.08 M sodium succinate dibasic hexahydrate (Sigma-Aldrich, Carlsbad, CA). All the slides were stained together in the same jar and then washed in physiological saline (0.85% NaCl in H_2_O), fixed in neutral buffered 10% formalin (Sigma-Aldrich, Carlsbad, CA), incubated in 15% EtOH for 5 min, and finally mounted with a water-soluble medium (DAKO fluorescent mounting medium; Agilent, Santa Clara, CA) and sealed with acrylic nail polish. The slides were scanned using a TissueFAXS 200 with TissueFAXS Viewer 1016 software (TissueGnostics, Tarzana, CA). ImageJ was used to measure the pixel intensities of the entire tibialis anterior and soleus sections. A region of interest was selected to include the whole muscle of interest (and exclude adjacent muscles, which were sectioned along with the muscles of interest to preserve tissue structure), the background signal was subtracted, and the image was transformed to 8-bit grey scale. Histograms of the pixel intensity distribution were obtained for 2 sections/mouse by a blinded operator using the Analysis<Histogram function, and the data were averaged for 3 mice/group.

### mtDNA mutation evaluation

Frozen muscle samples were homogenized in liquid nitrogen in a ceramic mortar using a ceramic pestle. An organic solvent extraction-based DNA preparation method (PCIAA) was used for DNA extraction, as previously described as one of the best methods for mtDNA isolation [80]. mtDNA damage was estimated via long-range QPCR method, based on a previously described protocol [81]. Amplification of a 10Kb long mitochondrial fragment was compared to that of a short 117bp mitochondrial fragment to estimate lesion frequency. Long fragment primers – Fw: GCCAGCCTGACCCATAGCCATAATAT; Rev: GAGAGATTTTATGGGTGTAATGCGG. Short fragment primers – Fw: CCCAGCTACTACCATCATTCAAGT; Rev: GATGGTTTGGGAGATTGGTTGATGT. For long fragment PCR amplification, a 20 μl reaction containing 8 ng template, 100 nM primers, 200 µM dNTPs, 3% DMSO, and 0.4 U Phusion High Fidelity Polymerase (New England Biolabs, Thermo Scientific®, Ipswich, MA) was prepared, and following an initial 98°C 3 min step, reactions proceeded for 20 cycles of 98°C for 10 sec, 58°C for 15 sec and 72°C for 5 min and 30 sec, followed by a final step at 72°C for 10 min. For short fragment PCR amplification, a 20 μl reaction containing 8 ng template, 200 nM primers, 200 µM dNTPs and 0.4 U Phusion High-Fidelity DNA Polymerase (New England Biolabs, Ipswich, MA) was prepared, and following an initial 98°C 3 min step, reactions proceeded for 18 cycles of 98°C for 10 sec, 58°C for 15 sec and 72°C for 15 sec, followed by a final step at 72°C for 10 min. Quantification was performed using a Pico488 DNA quantification kit according to the manufacturer’s instructions (Lumiprobe Corporation, Cockeysville, MD).

### Statistical analysis

Statistical significance was evaluated using GraphPad Prism. The t-test or one-way-ANOVA Tukey test was used to compare two or more than two groups, respectively. *p ≤ 0.05, **p ≤ 0.01, ***p ≤ 0.001, ****p ≤ 0.0001

## SUPPLEMENTARY MATERIAL

Figure S1

Figure S2

Figure S3

Figure S4

Figure S5

Figure S6

Figure S7

Figure S8
