# Supplementary material for: Oxidative muscles have better mitochondrial homeostasis than glycolytic muscles throughout life and maintain mitochondrial function during aging
Source: Aging (Albany NY). 2018 Nov 18;10(11):3327–52. doi: 10.18632/aging.101643 (PMC6286850; doi:10.18632/aging.101643)
Supplement: Figure S1 [file aging-10-101643-s001.pdf]

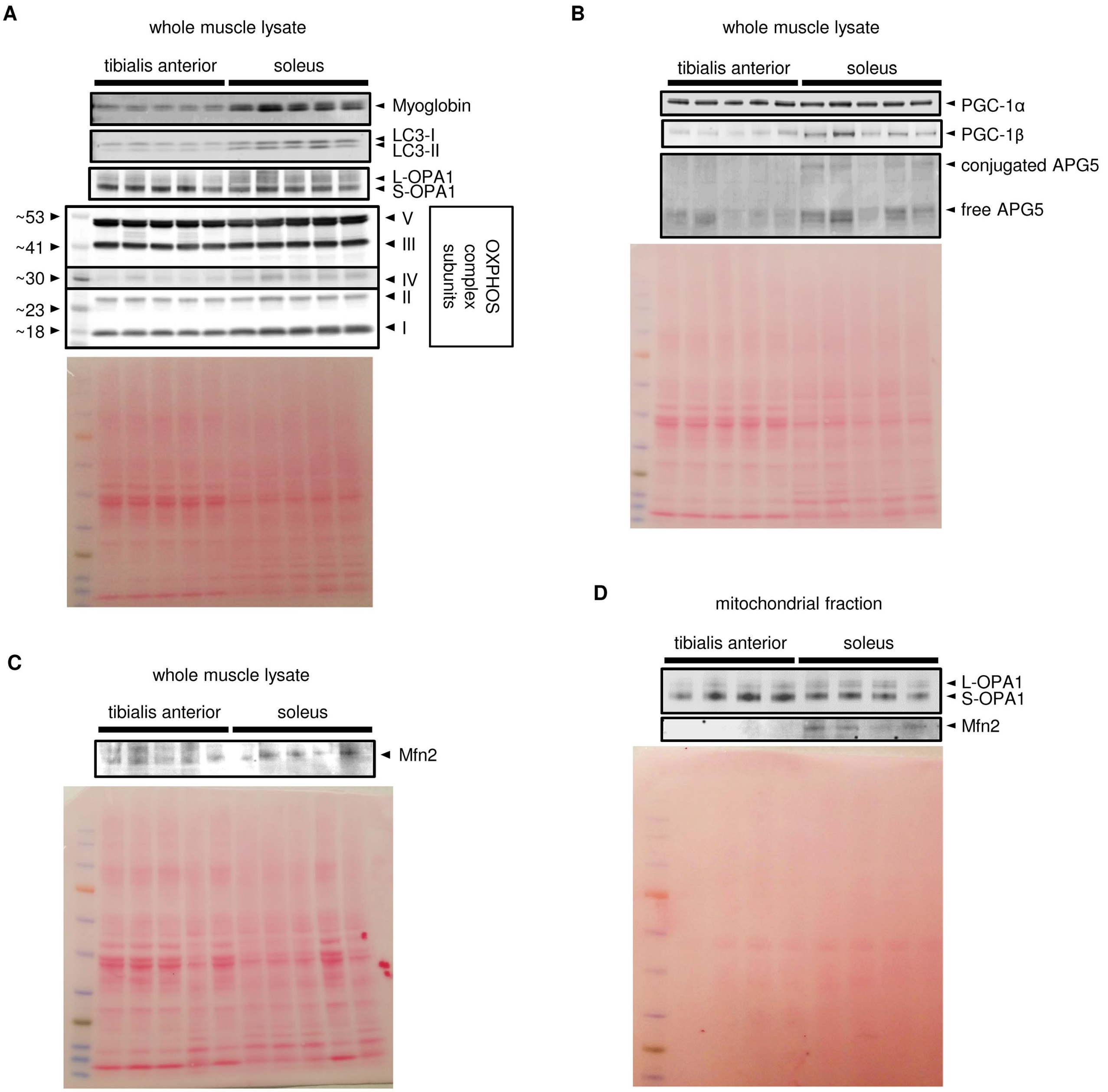

Figure S1. In young mice, the soleus is more oxidative than the tibialis anterior, and expresses higher levels of mitochondrial biogenesis, fission/fusion and autophagy markers. Markers of oxidative metabolism (myoglobin and representative OXPHOS complex subunits), mitochondrial biogenesis (PGC-1α, PGC-1β), fusion/fission (Mfn2, short (S) and long (L)-OPA1), and autophagy (LC3-II/I and APG5) were assessed in whole muscle lysates (**A-C**) and mitochondrial fractions (**D**) of tibialis anterior and soleus muscles from young (3 mo) mice. Ponceau-stained blots were used for normalization; the entire Ponceau lane was quantified for each sample. Representative blots are shown (A-C, 3 independent technical replicates of 5 mice/group; D, 4 mice/group).
