# Supplementary material for: Oxidative muscles have better mitochondrial homeostasis than glycolytic muscles throughout life and maintain mitochondrial function during aging
Source: Aging (Albany NY). 2018 Nov 18;10(11):3327–52. doi: 10.18632/aging.101643 (PMC6286850; doi:10.18632/aging.101643)
Supplement: Figure S2 [file aging-10-101643-s002.pdf]

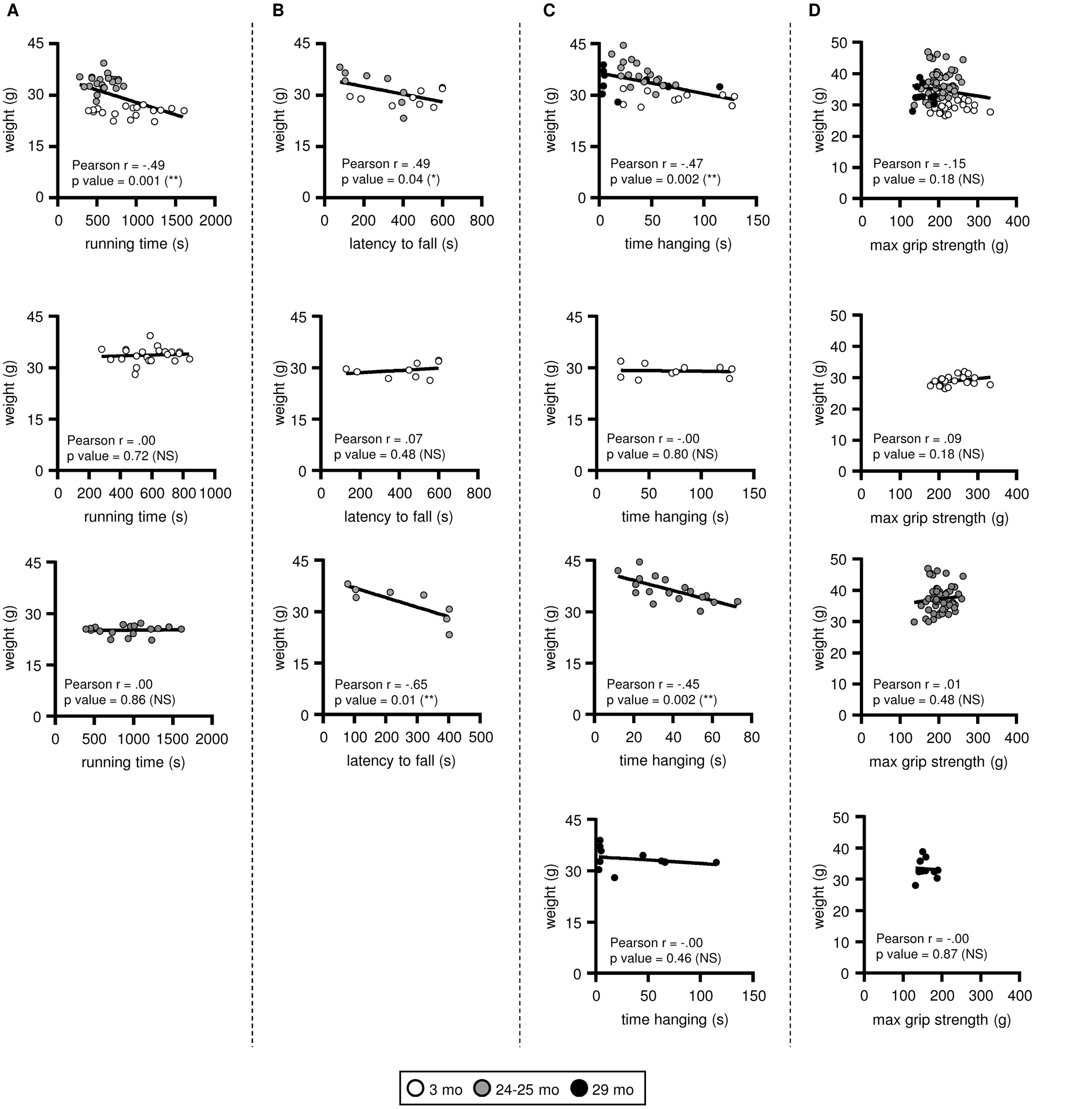

Figure S2. Correlation between body weight and treadmill, rotarod, hang-wire and grip strength test performance. Exercise performance and muscle strength were evaluated in young (3 mo) and old (24-25 and 29 mo) mice and correlated with body weight. (**A**) Time to exhaustion on the treadmill; 3 mo (n=20), 24 mo (n=24). (**B**) Latency to fall from the rotarod; 3 mo (n=9), 24 mo (n=24). (**C**) Time spent hanging in the hang-wire test; 3 mo (n=11), 24 mo (n=18), 29 mo (n=11). (**D**) Four-limb grip strength test; 3 mo (n=20), 24 mo (n=26), 24 mo (n=24), 29 mo (n=11).
