# Supplementary material for: Oxidative muscles have better mitochondrial homeostasis than glycolytic muscles throughout life and maintain mitochondrial function during aging
Source: Aging (Albany NY). 2018 Nov 18;10(11):3327–52. doi: 10.18632/aging.101643 (PMC6286850; doi:10.18632/aging.101643)
Supplement: Figure S4 [file aging-10-101643-s004.pdf]

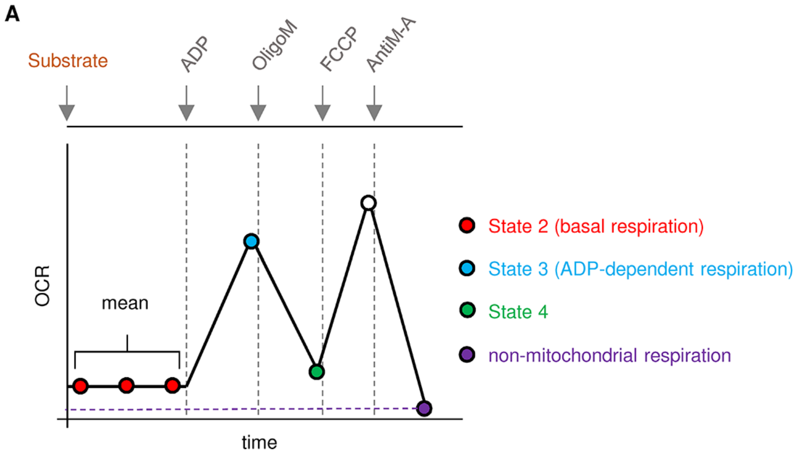

METHOD	
State 2 (basal respiration)	= mean of 3 basal state OCR measurements
State 3 (ADP-dependent respiration)	= OCR after ADP injection
State 4	= OCR after Oligomycin M injection (inhibition of ATP synthase)
Respiratory Control Index (RCI)	= State 3/State 4

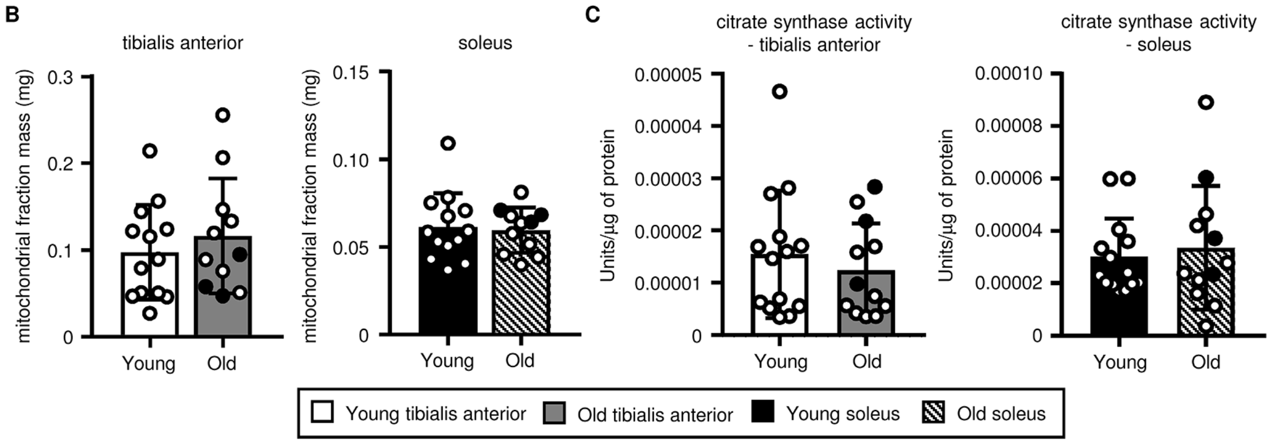

Figure S4. Respirometry analysis and assessment of mitochondrial aging. Seahorse assays were performed to assess mitochondrial function. (**A**) Experimental set up for respirometry assessment using pyruvate, succinate or palmitoylcarnitine as substrates. OCR: oxygen consumption rate; OligoM: oligomycin; AntiM-A: antimycinA. State 2 (basal), State 3 (ADP-dependent), and State 4 (oligomycin-insensitive) respiration, and the Respiratory Control Index (RCI) were calculated as indicated. (**B**) The mass of the mitochondrial fractions isolated from the whole tibialis anterior and soleus muscles of young (3 mo, n=13) and old (28-29 mo, n=11) mice was calculated using a DC assay. (**C**) The citrate synthase activity per microgram of the mitochondrial fraction from the tibialis anterior and soleus muscles of young (3 mo, n=13) and old (28-29 mo, n=12) mice was evaluated using an assay kit.
Congenic CD45.2 and CD45.1 old mice were used (b-c; young mice were all CD45.2): white dots = CD45.2, black dots = CD45.1.
