# Supplementary material for: Oxidative muscles have better mitochondrial homeostasis than glycolytic muscles throughout life and maintain mitochondrial function during aging
Source: Aging (Albany NY). 2018 Nov 18;10(11):3327–52. doi: 10.18632/aging.101643 (PMC6286850; doi:10.18632/aging.101643)
Supplement: Figure S5 [file aging-10-101643-s005.pdf]

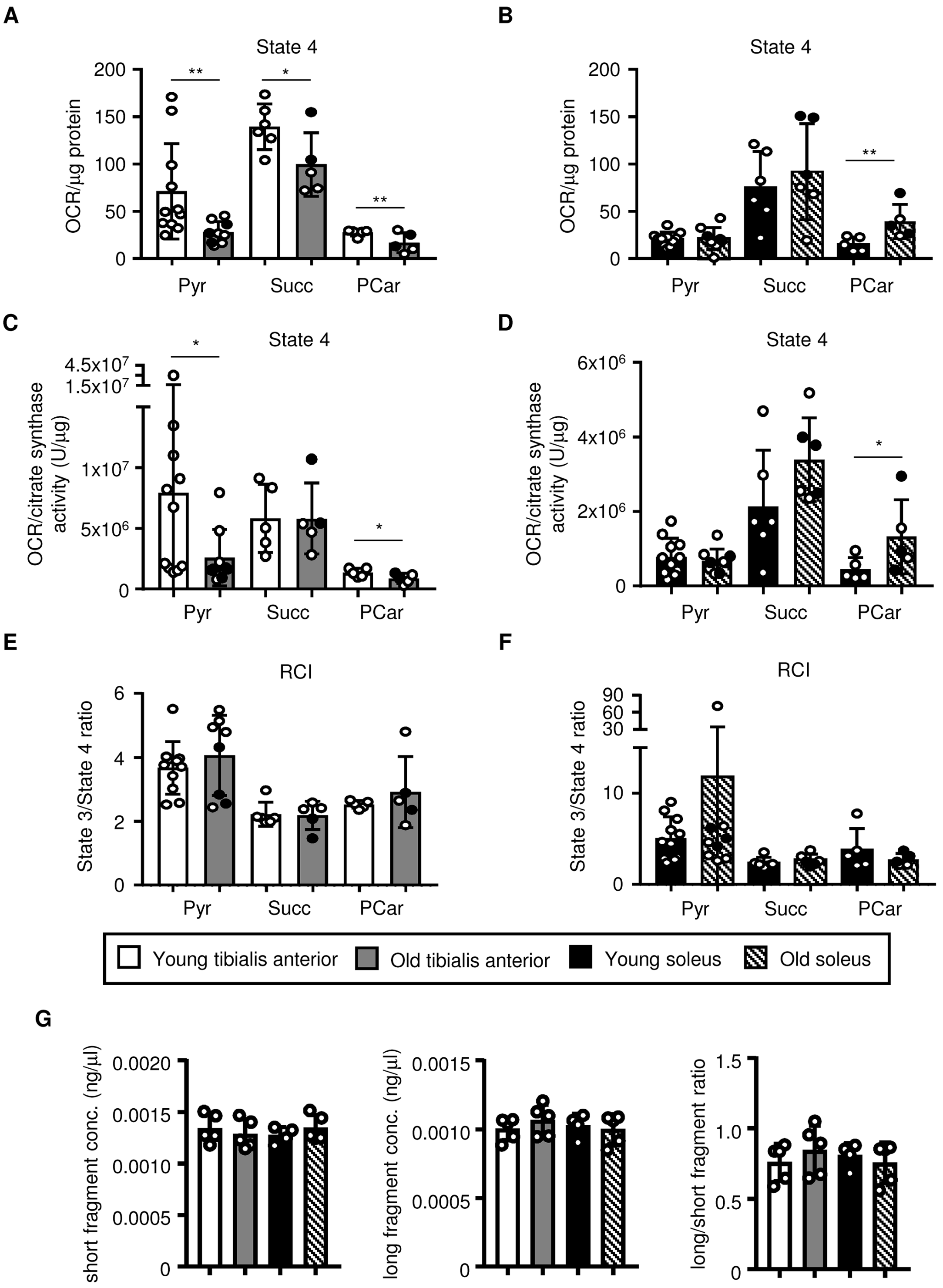

Figure S5. Respirometry analysis and assessment of mitochondrial aging. (**A-F**) State 4 (oligomycin-insensitive) mitochondrial respiration normalized to either the protein content of the mitochondrial fraction (**A-B**) or citrate synthase activity/µg of protein (**C-D**), and the Respiratory Control Index (RCI = State 3/State 4 ratio, which is the same using both normalization methods; (**E-F**) were assessed in the tibialis anterior and soleus muscles of young (3 mo, n=13) and old (28-29 mo, n=11) mice. Congenic CD45.2 and CD45.1 old mice were used (young mice were all CD45.2): white dots = CD45.2, black dots = CD45.1. (**G**) Quantitative PCR was used to assess mtDNA mutation. A long fragment covering a mtDNA locus that is highly susceptible to mutation was amplified and quantified to assess intact mtDNA (left). Total mtDNA was evaluated by amplifying a short fragment of mtDNA in an un-mutated locus (middle). The long/short fragment ratio (fraction of mtDNA that is intact) was then calculated (right). *p ≤ 0.05, **p ≤ 0.01
