# Supplementary material for: Oxidative muscles have better mitochondrial homeostasis than glycolytic muscles throughout life and maintain mitochondrial function during aging
Source: Aging (Albany NY). 2018 Nov 18;10(11):3327–52. doi: 10.18632/aging.101643 (PMC6286850; doi:10.18632/aging.101643)
Supplement: Figure S7 [file aging-10-101643-s007.pdf]

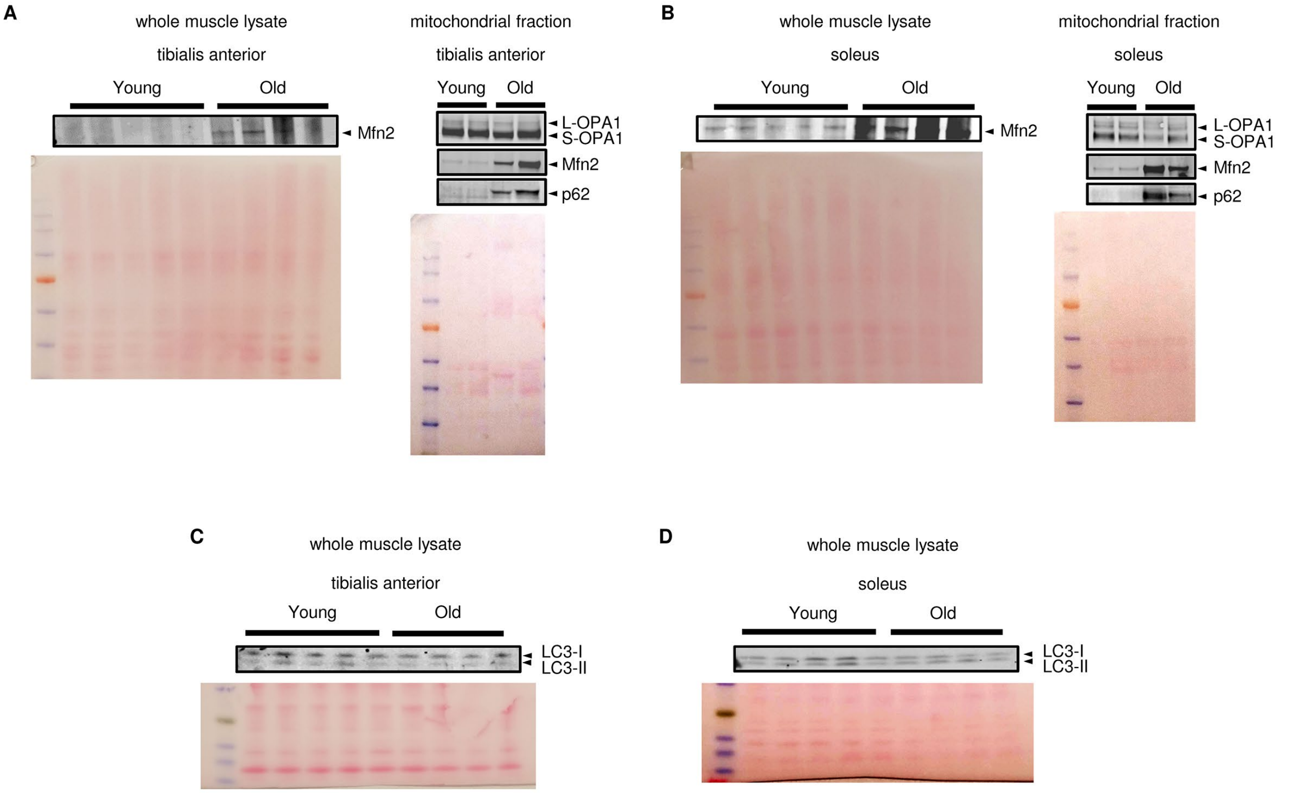

Figure S7. Upon aging, the tibialis anterior and soleus undergo similar changes in mitochondrial biogenesis, fission/fusion, disposal and autophagy marker expression. Markers of mitochondrial fusion/fission and autophagy were assessed in whole muscle lysates and mitochondrial fractions (as indicated) of tibialis anterior and soleus muscles from young (3 mo) and old (28-29 mo) mice. Ponceau-stained blots were used for normalization; the entire Ponceau lane was quantified for each sample. Representative blots are shown (3 independent technical replicates of 4-5 mice/group).
