# Supplementary material for: Oxidative muscles have better mitochondrial homeostasis than glycolytic muscles throughout life and maintain mitochondrial function during aging
Source: Aging (Albany NY). 2018 Nov 18;10(11):3327–52. doi: 10.18632/aging.101643 (PMC6286850; doi:10.18632/aging.101643)
Supplement: Figure S8 [file aging-10-101643-s008.pdf]

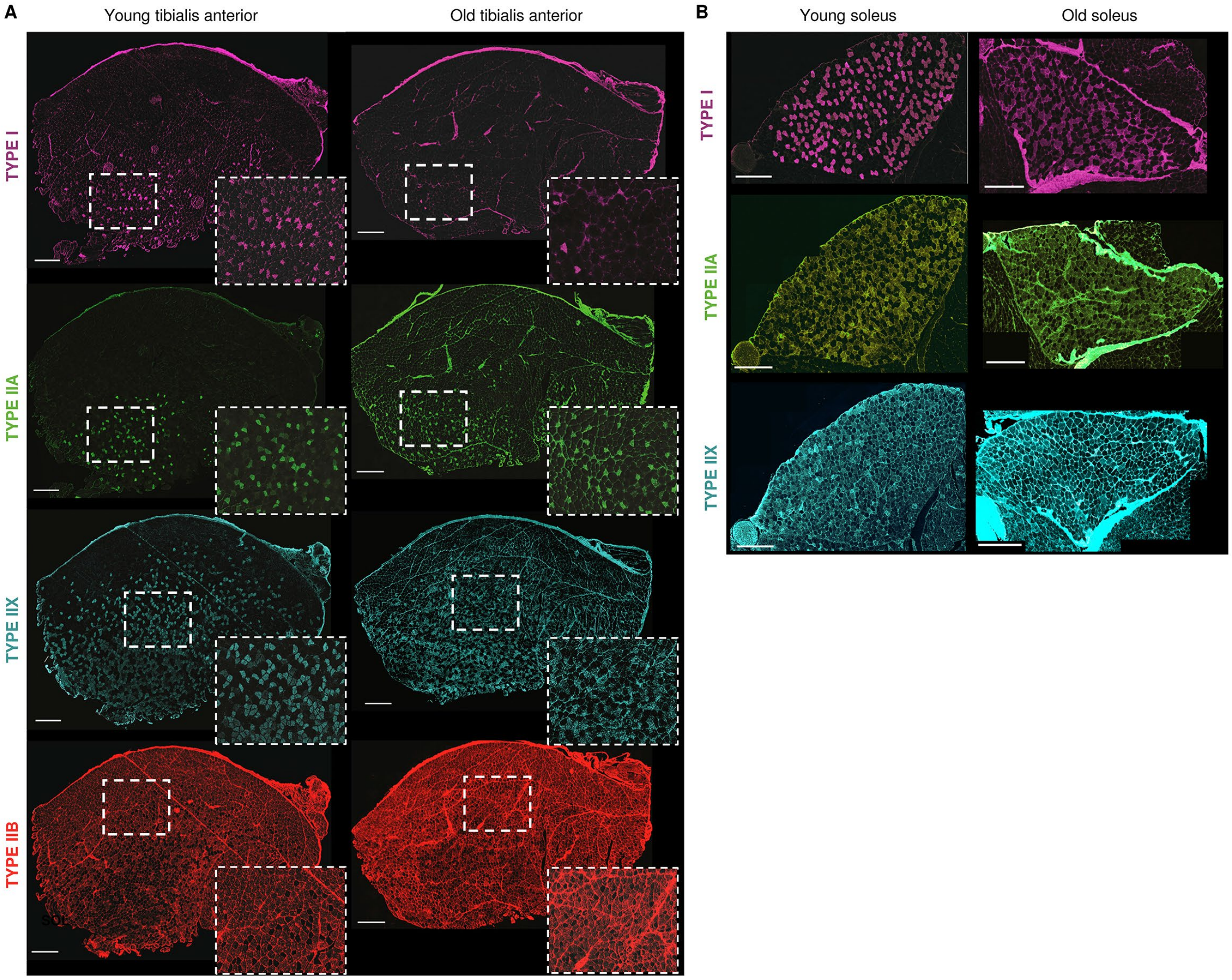

Figure S8. Myosin heavy chain expression by young and old tibialis anterior and soleus muscles. Cryosections of young and old tibialis anterior (**A**) and soleus (**B**) muscles were stained with anti-mouse type I, IIA, IIX and IIB myosin heavy chain (MyHC) antibodies to identify muscle fiber types. Representative images are shown. Scalebar: 400 µm.
